# Long-term prognosis and DNA damage status after oral mucosal epithelial cell sheet transplantation following esophageal endoscopic submucosal dissection for squamous cell carcinoma: A case series

**DOI:** 10.1016/j.reth.2024.08.007

**Published:** 2024-08-10

**Authors:** Yasuhiro Maruya, Yuko Akazawa, Kiyuu Norimatsu, Yerkezhan Sailaubekova, Nazigul Zhumagazhiyeva, Shinichiro Kobayashi, Miki Higashi, Keiichi Hashiguchi, Naoyuki Yamaguchi, Masahiro Nakashima, Kazuhiko Nakao, Kengo Kanetaka, Susumu Eguchi

**Affiliations:** a Tissue Engineering and Regenerative Therapeutics in Gastrointestinal Surgery, Nagasaki University Graduate School of Biomedical Sciences, Nagasaki, Japan; b Department of Surgery, Nagasaki University Graduate School of Biomedical Sciences, Nagasaki, Japan; c Department of Histology and Cell Biology, Nagasaki University Graduate School of Biomedical Sciences, Nagasaki, Japan; d Department of Tumor and Diagnostic Pathology, Atomic Bomb Disease Institute, Nagasaki University, Nagasaki, Japan; e Department of Gastroenterology and Hepatology, Nagasaki University Graduate School of Biomedical Sciences, Nagasaki, Japan

**Keywords:** Cell sheet, Esophageal squamous cell carcinoma, 53BP1

## Abstract

Autologous oral mucosal epithelial cell sheet (AOMECS) transplantation has recently been applied in human patients to prevent postprocedural stenosis following endoscopic submucosal dissection (ESD) for esophageal squamous cell carcinoma. However, the long-term safety of AOMECS transplantation remains unclear. We evaluated the long-term outcomes of 10 patients who participated in a clinical trial of AOMECS transplantation after esophageal ESD. Additionally, we assessed the local DNA damage response in the esophageal epithelium using p53 binding protein 1 (53BP1) immunofluorescence in post-AOMECS biopsy specimens. The median follow-up period was 118.5 months (range: 46–130 months). Two patients developed primary esophageal cancer near the AOMECS site and successfully underwent additional ESD. One patient developed lymph node metastasis and underwent chemotherapy. None of the patients died from the original disease, although one patient died from unrelated causes. The rate of abnormal 53BP1 nuclear foci, indicative of increased genome instability, increased with the progression of neoplasia in patients post AOMECS. Our case series suggests that AOMECS transplantation provides an acceptable long-term prognosis and 53BP1 foci may serve as a useful marker for assessing DNA instability in the post-AOMECS esophageal epithelium.

## 1 Introduction

Endoscopic submucosal dissection (ESD) is a well-established method that enables *en bloc* removal of superficial esophageal squamous cell carcinomas (SCC) [1]. However, one of the major obstacles of esophageal ESD is the development of esophageal strictures, which largely affect a patient's quality of life [1]. Oral administration or local injection of steroid therapy has been reported to be effective in preventing post-esophageal ESD stenosis, but has potential side effects, such as immunosuppression, diabetes, and osteoporosis.

Cell sheet technology has recently been successfully applied to regenerative medicine. Endoscopic transplantation of autologous oral mucosal epithelial cell sheet (AOMECS) has been reported to counteract post-ESD esophageal strictures [2]. Yamaguchi et al. [3] and the Tokyo Women's Medical University conducted a clinical study involving the fabrication and transport of cell sheets between a cell culture facility at the Institute of Advanced Biomedical Engineering and Science of Tokyo Women's Medical University and Nagasaki University Hospital, covering a distance of no less than 1200 km by airplane with a travel time of seven  hours. The cell sheets were endoscopically transplanted without sutures on the ulcer immediately after extensive ESD for superficial esophageal SCC. No short-term serious adverse events were observed in this study [3]. A phase I trial in Western countries confirmed the short-term safety of AOMECS transplantation for Barrett esophageal cancer [4]. However, long-term safety, including potential carcinogenesis after AOMECS transplantation, has not been examined. Moreover, an optimal method to monitor the quality of the epithelium after AOMECS transplantation should be developed to assess its long-term safety.

Genomic instability contributes to stepwise carcinogenesis, and its presence can be inferred from an increased nuclear foci of the DNA damage response protein p53 binding protein-1 (53BP1) [[5], [6], [7], [8]]. Previous immunofluorescence studies indicated alterations of the 53BP1 nuclear foci expression pattern, including increased number and size, during carcinogenesis [6,8]. In addition, since the DNA damage response occurs during cell arrest in a healthy state, the expression of 53BP1 in a proliferative state indicates genomic instability [9].

In the current study, we aimed to demonstrate the long-term clinical and histological outcomes of esophageal regeneration therapy using AOMECS transplantation. Furthermore, we examined the DNA damage response status using 53BP1 immunofluorescence.

## 2 Patients

A summary of patients is presented in Table 1. Ten Patients (median age, 65 years; range, 55–74 years) who underwent AOMECS transplantation after ESD for superficial esophageal SCC at Nagasaki University Hospital from July 2013 to October 2014 [3] were retrospectively investigated for long-term prognosis and esophageal cancer recurrence after surgery (Table 1.) The initial median SCC tumor size was 35.5 mm (range, 25–78 mm). The median resected area was 2590 mm^2^ (range, 1350–7980 mm^2^). All cases achieved en bloc resection. The tumor invasion depth subclassification was T1s-EP in one patient, T1a-LPM in six patients, T1a-MM in two patients, and T1b-SM in one patient. En bloc resection was performed in all the 10 patients. Three ESD cases were determined as noncurative resection because of lymphatic invasion (case 1), lymphovascular invasion and positive vertical margins (case 7), and invasion of the submucosal layer (case 10), all of which were treated with additional chemoradiotherapy. The histological diagnosis and classification of the resected specimens were based on the Japanese Classification of Esophageal Cancer, 11th Edition [10].

**Table 1:** Characteristics of the patients who underwent ESD/AOMECS.

Cases	1	2	3	4	5	6	7	8	9	10
Age/Sex	55/M	68/M	73/M	58/M	67/M	56/M	63/M	72/M	62/F	74/M
Histology	SCC	SCC	SCC	SCC	SCC	SCC	SCC	SCC	SCC	SCC
Tumor size (mm)	42	35	25	33	30	36	55	78	43	35
Resected area	4400	5200	1350	2530	1650	2200	4015	7985	2650	2070
Depth of tumor	LPM	LPM	LPM	EP	LPM	LPM	MM	MM	SM2	LPM
Lymphatic invasion	+	–	–	–	–	–	+	–	–	–
Vessel invasion	–	–	–	–	–	–	+	–	–	–
Positive vertical margin	–	–	–	–	–	–	+	–	+	–
Additional treatment	CRT	–	–	–	–	–	CRT	–	CRT	–
Observation period (months)	130	126	125	127	119	88	118	91	118	46
LNM recurrence	–	–	–	–	–	–	+	–	–	–
Neoplasm at AOMECS site	SCC in situ	SCC	IN	–	–	–	IN	–	–	–
Metachronous primary cancer	–	–	–	+	–	–	–	–	–	–
Additional treatment	ESD	ESD	–	ESD	–	–	CT	–	–	–
Other primary cancers	Pharynx /Lung	–	Pharynx	Pharynx	–	–	Lung	Stomach	–	Colon /Lung
Prognosis	Alive	Alive	Alive	Alive	Alive	Alive	Alive	Alive	Alive	Deceased

ESD, endoscopic submucosal dissection; AOMECS, autologous oral mucosal epithelial cell sheet; SCC, squamous cell carcinoma; EP, invasion of epithelium; LPM, invasion through the basement membrane to the lamina propria mucosa; MM, invasion to the muscularis mucosa; SM2, submucosal invasion by > 200 μm; LNM, lymph node metastasis; CRT, chemoradiotherapy; CT, chemotherapy; IN, intraepithelial neoplasia.

## 3 Summary of outcomes after AOMECS

A summary of patients is presented in Table 1. The median follow-up period after ESD and AOMECS was 118.5 months (range, 46–130 months). Serial upper endoscopic and CT examinations were performed in all patients, and biopsy specimens were obtained if precancerous lesions were suspected. One patient (case 10) died of primary lung cancer and nine other patients survived. One patient (case 7) who developed lymph node metastasis after esophageal ESD and AOMECS underwent chemotherapy and survived during the 100-month follow-up period. One patient developed SCC (case 4) in the esophagus at a location not affected by AOMECS. One patient (case 1) showed carcinoma in situ, and one developed SCC (case 2) in proximity to the ESD scar at the site of AOMECS. These cases were both considered to be metachronous cancers rather than local recurrences as the margins of both carcinomas were negative. Primary carcinoma outside the esophagus was seen in the majority of patients, which consisted of three pharyngeal cancers, two lung cancers, and one stomach cancer.

**Fig. 1:**
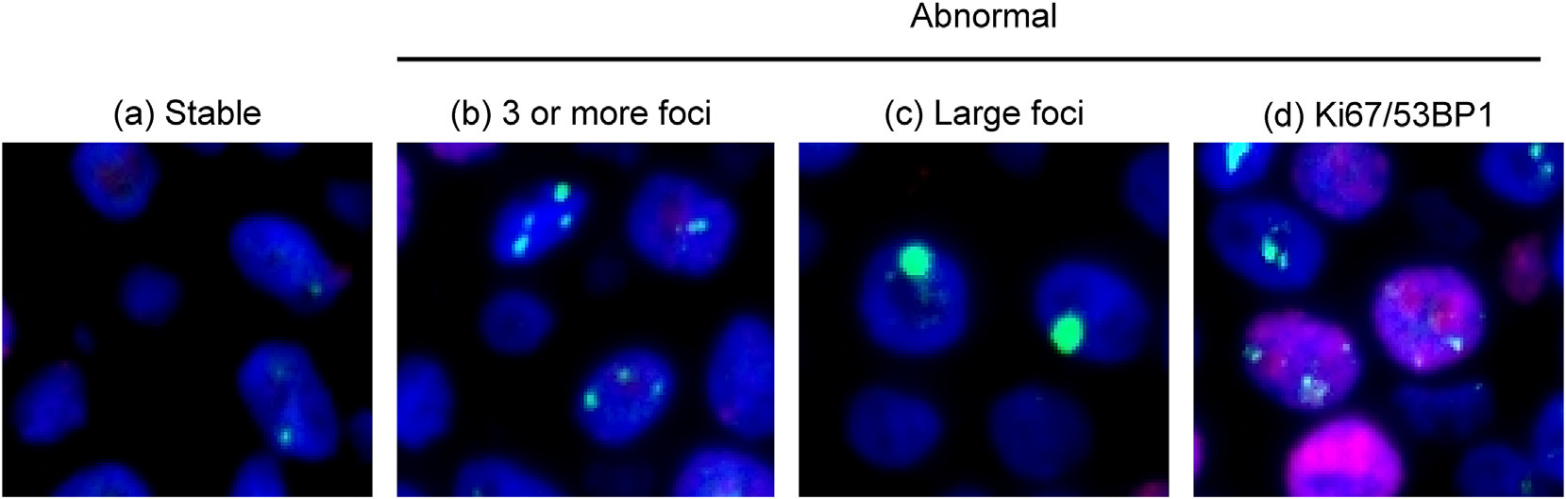
**Classification of p53-binding protein 1 (53BP1) expression in esophageal biopsy specimens from post-ESD scars**. (a) Normal/stable pattern (≤2 nuclear foci), Abnormal patterns show either (b) more than three nuclear foci, (c) large foci (>1 μm in diameter), or (d) colocalization of Ki67/53BP1.

Table 2 shows details of esophageal biopsy samples from the post-ESD scar with AOMECS transplantation. Of the 10 patients, four underwent biopsy with suspected carcinomas at the ESD/AOMECS site, whereas six patients did not undergo biopsy. Detailed descriptions of the biopsy results and DNA damage status are shown in Table 2. 53BP1 expression in ≥3 foci and/or foci >1.0 μm in diameter was defined as abnormal (Fig. 1) [11]. The colocalization of 53BP1 with Ki67, which indicates 53BP1 foci expression in a proliferative state, was also categorized as abnormal foci [9] based on our previous studies (Fig. 1).

**Table 2:** 53BP1 expression, number of Ki67-positive cells, and percentage of 53BP1 focus-containing nuclei in Ki67-positive cells in esophageal biopsy tissues from post-ESD scars with AOMECS transplantation.

Case number	Months from AOMECS	Histological diagnosis	Abnormal foci, %	Large forci, %	53BP1/Ki67 colocalization,%
1	6	SCC in situ	0.4	2.79	1.1
2	30	Intraepithelial neoplasia	0.71	5.23	1.3
2	35	Nonneoplastic	1.01	0	0
2	48	Intraepithelial neoplasia	2.89	6.87	1.81
2	54	Squamous cell carcinoma	4.97	25.9	5.4
3	49	Intraepithelial neoplasia	1.42	7.72	2.64
3	53	Intraepithelial neoplasia	11.6	11.1	13.4
7	3	Nonneoplastic	2.93	7.71	4.52
7	8	Nonneoplastic	5.88	3.78	2.1
7	47	Intraepithelial neoplasia	1.22	7.11	0.81

53BP1, p53 binding protein 1; ESD, endoscopic submucosal dissection; AOMECS, autologous oral mucosal epitherial cell sheet; SCC, squamous cell carcinoma.

## 4 Details of each case

**Fig. 2:**
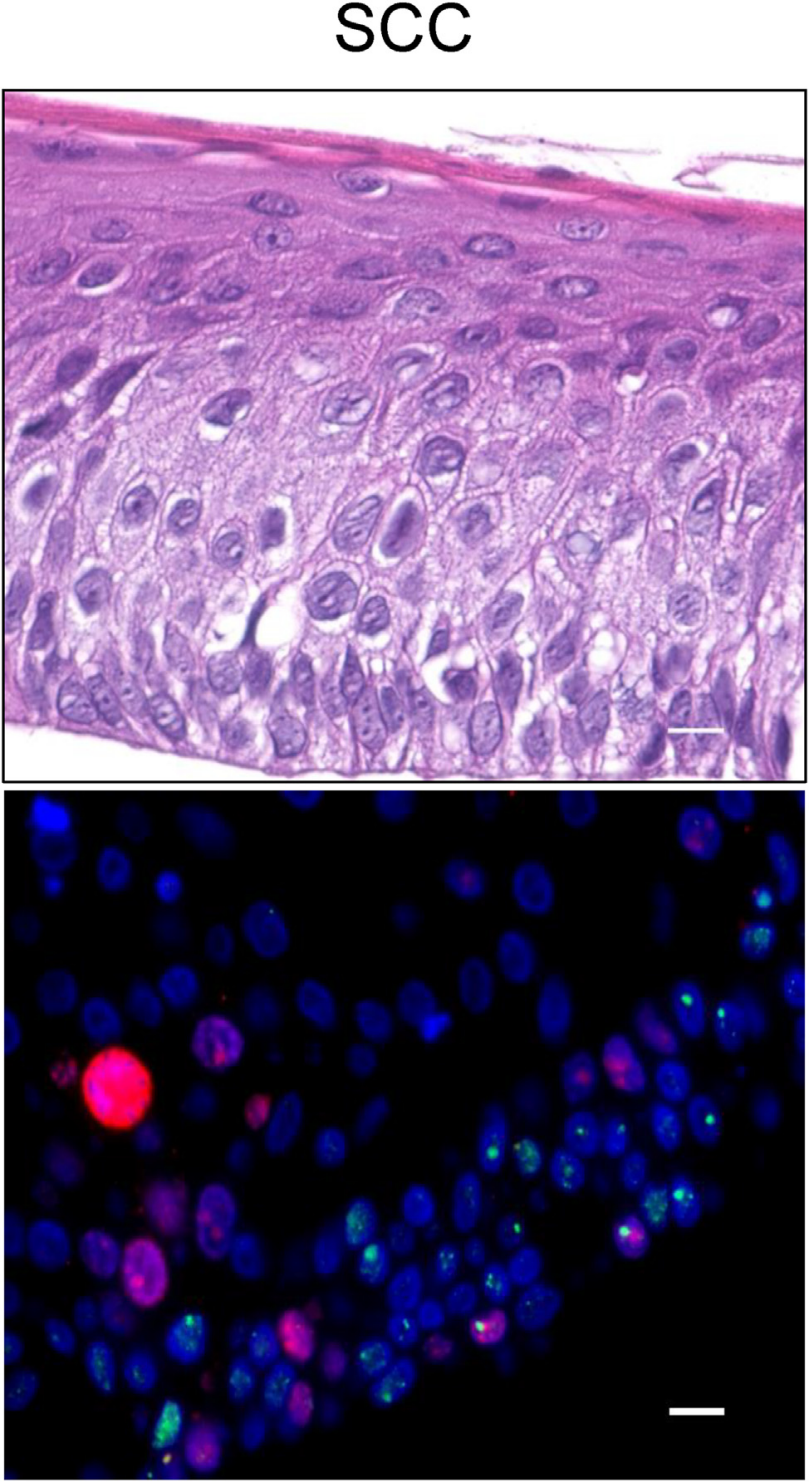
**HE and double-label immunofluorescence with 53 BP1 and Ki67 in case 1**. The biopsy was taken 6 months after AOMECS. Blue, nucleus; red, Ki67; green, Ki67. Original magnification, 100×. Scale bars indicate 10 μm.

Case 1: The patient was a 55-year-old male at the time of ESD/AOMECS. SCC was diagnosed 6 months after ESD/AOMECS in situ. Additional ESD was performed, which resulted in curative resection. Images of H and E staining as well as 53BP1 are shown in Fig. 2. The patient later developed advanced primary pharyngeal SCC, which was treated with total laryngectomy, left thyroid lobectomy, bilateral neck lymph node dissection, and tracheostomy at 54 months. Sixteen months after the initial surgery for pharyngeal SCC, the patient underwent partial thoracoscopic resection of the lower lobe for metastatic lung carcinoma. The patient was well without recurrence after 130 months of follow-up.

**Fig. 3:**
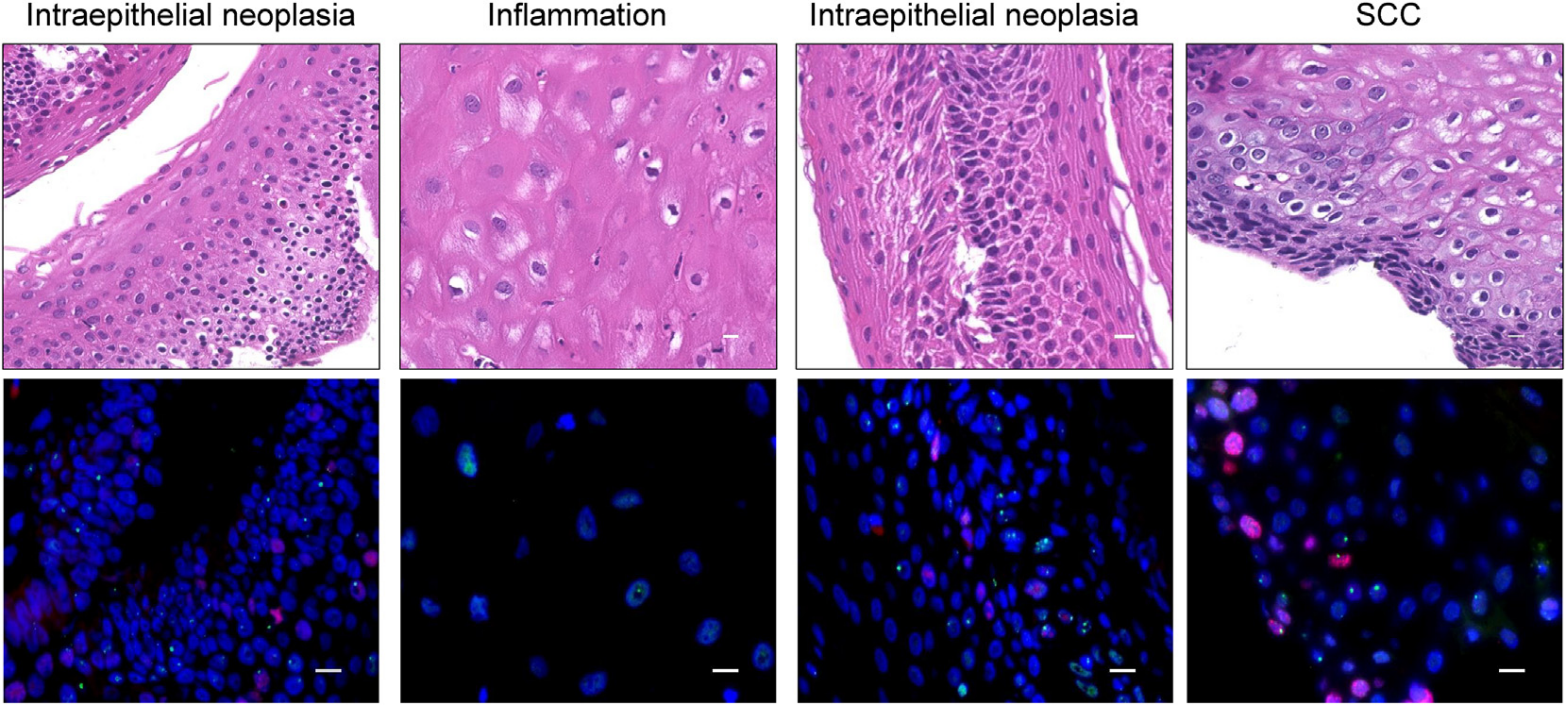
**HE and double-label immunofluorescence with 53 BP1 and Ki67 in case 2**. The biopsy was taken 30 (left panel), 34 (left middle panel), 48 (right middle panel), and 54 (right panel) months after AOMECS. Blue, nucleus; red, Ki67; green, Ki67. Original magnification, 100×. Scale bars indicate 10 μm.

Case 2: The patient was a 68-year-old male. He underwent serial biopsy at 30,35,48, and 54 months at the AOMECS site during follow-up (Table 2). No malignancy was suspected in the first few years. An initial biopsy after AOMECS was performed 30 months after the diagnosis of intraepithelial neoplasia (Fig. 3). The follow-up biopsy results were non-neoplastic at 35 months. The next follow-up biopsy at 48 months showed intraepithelial neoplasia. A biopsy at 54 months showed progression to SCC. 53BP1 analysis showed an increase in abnormal foci and large foci and 53BP1/Ki67 co-localization in SCC compared to intraepithelial neoplasia (Table 2). Additional ESD was performed, and no recurrence was observed during a follow-up period of 126 months.

**Fig. 4:**
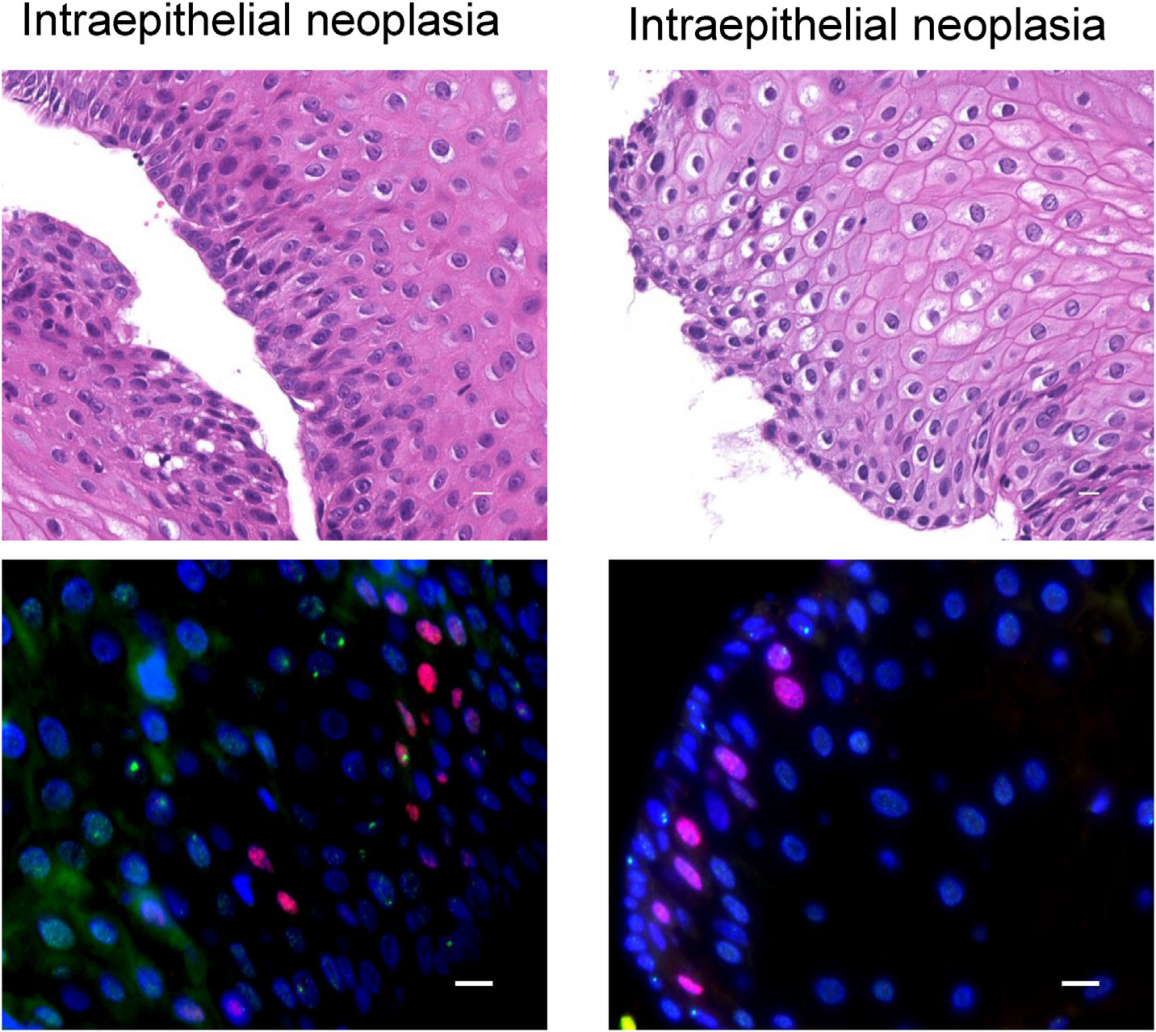
**HE staining and double-label immunofluorescence staining with 53 BP1 and Ki67 in case 3**. The biopsy was taken 49 (left panel) and 54 months (right panel) after AOMECS. Blue, nucleus; red, Ki67; green, Ki67. Original magnification, 100×. Scale bars indicate 10 μm.

Case 3: The patient was a 73-year-old male. During the endoscopic period, SCC was found in the pharynx at 43 and 51 months, both of which were successfully treated by ESD. The patient underwent follow-up esophageal biopsy twice at 49 and 53 months, both presenting with intraepithelial neoplasia (Fig. 4). The patient was followed up because there were no findings suspicious for malignancy on subsequent periodic endoscopic examinations. Although 53BP1 examination in both biopsies showed an increase in 53BP1 nuclear foci during the 4 month-follow period, no progression to malignancy was observed in the esophagus (Fig. 4). The patient survived without recurrence after 118 months.

Case 4: The patient was a 58-year-old male. During the follow-up endoscopy series, no biopsy was obtained from the AOMECS. However, the patient developed metachronous squamous neoplasms outside the AOMECS area in the esophagus at 6 and 18 months and underwent additional ESDs. The patient developed pharyngeal SCC during follow-up and underwent total pharyngolaryngoesophagectomy at 81 months. The ESD/AOMECS sites remained intact throughout the follow-up period. The patient was alive without recurrence at 127 months.

Case 5: The patient was a 67-year-old male with ESD/AOMECS. During the follow-up endoscopy series, the ESD/AOMECS site remained intact and a biopsy was not obtained. The patient survived for 119 months without esophageal malignancies.

Case 6: A 56-year-old male. During the follow-up endoscopy series, no biopsy was obtained from AOMECS. The patient followed up for 88 months without recurrence or metachronous cancer.

**Fig. 5:**
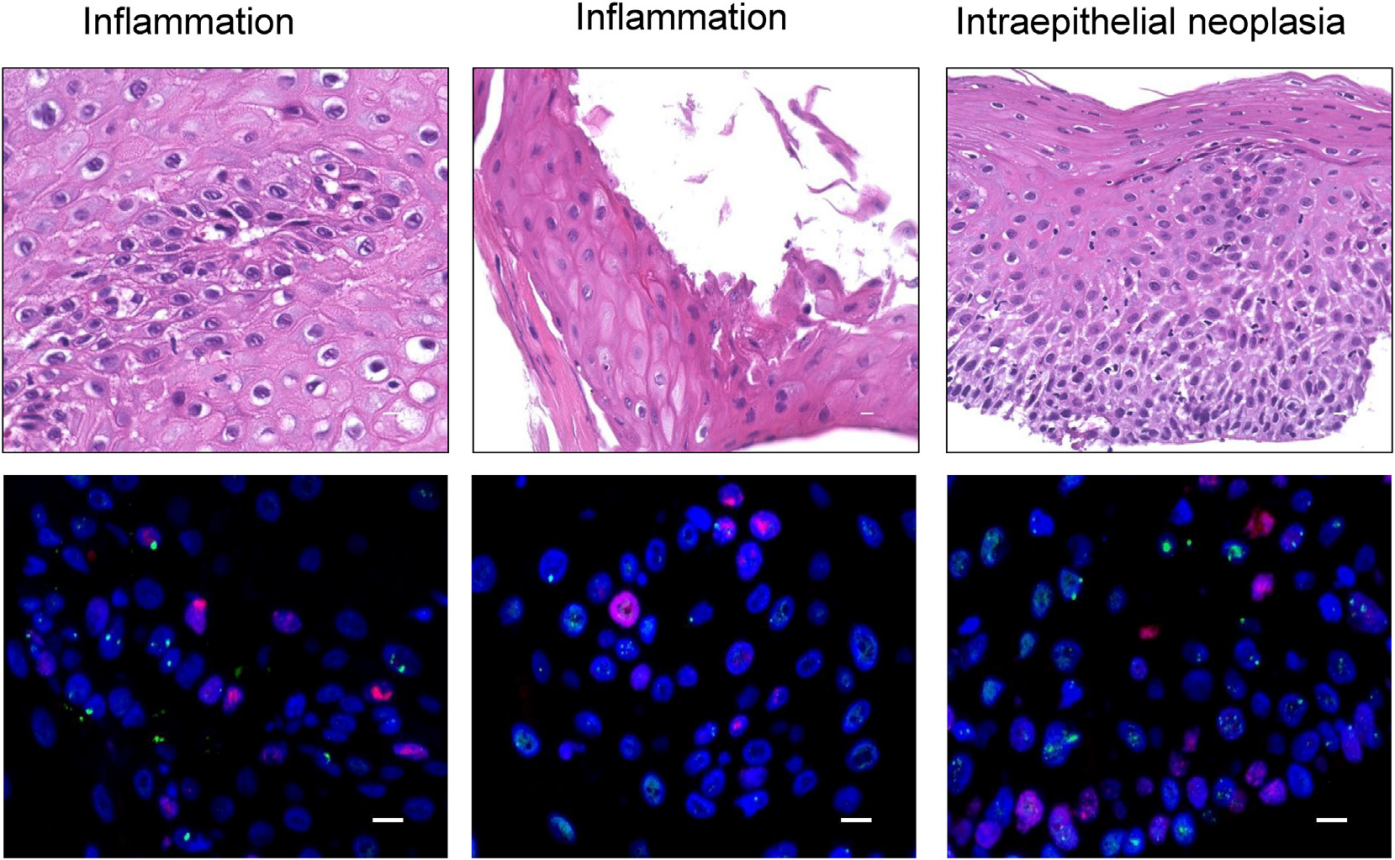
**HE staining and double-label immunofluorescence staining with 53 BP1 and Ki67 in case 3**. The biopsy was taken 3 (left panel), 8 (middle panel), and 47 (right panel) months after AOMECS. Blue, nucleus; red, Ki67; green, Ki67. Original magnification, 100×. Scale bars indicate 10 μm.

Case 7: The patient was a 63-year-old male. At the first ESD/AOMECS, pathological examination revealed a positive vertical margin with lymphovascular invasion of the tumor. The patient was strictly followed up by CT and later confirmed to have developed lymph node metastasis at 52 months, which was managed with chemotherapy. The patient underwent serial endoscopy. The follow-up biopsy of the ESD/AOMECS site demonstrated intraepithelial neoplasia (Fig. 5). 53BP1 analysis of the epithelium showed prominent 53BP1/Ki67 co-localization at 3 months, which later subsided in the biopsy performed at 8 and 47 months. This lesion was followed-up. The patient developed primary lung carcinoma and underwent thoracoscopic lung resection at 79 months. No further recurrence was observed in the esophagus or other organs during the follow-up period of 118 months.

Case 8: The patient was a 72-year-old male. Gastric adenocarcinoma was detected during follow-up endoscopy at month 34,66, and 70 months, all of which were successfully treated with ESD. ESD/AOMECS site did not show any suspicious lesions during 91 months follow-up.

Case 9: The patient was a 62-year-old female. Histological examination revealed positive vertical margins. The patient underwent conformal radiotherapy (CRT). Follow-up endoscopy showed no signs of malignancy, and a biopsy was not performed.

Case 10: The patient was a 74-year-old male. A follow-up endoscopy revealed no signs of malignancy. Twenty-six months after the AOMECS transplantation, thoracoscopic lower lobectomy was performed for lung cancer. The patient underwent reduced surgery owing to an underlying history of interstitial pneumonia. The patient eventually died of metastatic brain tumors at 46 months.

## 5 Discussion

Our pilot study showed that ESD/AOMECS resulted in favorable long-term prognosis, none of the patients died of the original disease, and all the locally recurrent or metastatic esophageal SCC cases were managed with follow-up treatment. In our study in 10 cases, curative resection was achieved in eight of the 10 patients (80%). According to Yeh et al., the mean curative resection rates for general esophageal ESD are 97.1% and 92.0%, respectively [12]. The overall local recurrence, metachronous recurrence, and lymph node/distance rates of ESD among all included studies were 1.8%, 8.5%, and 3.3%, respectively [12]. The lesions that require AOEMCS are generally large, which may have resulted in a relatively lower curative rate compared to that in general studies. Notably, seven of the ten patients developed primary tumors outside of AOMECS. Esophageal SCC is highly associated with alcohol consumption and smoking, which are risk factors for other primary cancers. Thus, regardless of AOMECS, patients with esophageal SCC should be followed up frequently.

Unlike other fields of regenerative medicine applications (e.g., heart, cornea, cartilage, etc.) [[13], [14], [15], [16], [17]], esophageal regenerative medicine using oral mucosal epithelial cell sheets enables real-time analysis of long-term safety in terms of carcinogenicity because endoscopic observation and biopsy of tissue over time is possible. The ability of AOMECS transplantation to prevent strictures after ESD for esophageal cancer is interpreted as a paracrine effect. Based on previous reports, cytokines and exosomes are released from AOMECS to promote wound healing and inhibit excessive fibrosis, which causes stricture [18,19]. For instance, the level of vascular endothelial growth factor (VEGF) in the supernatant culture medium was observed to increase during AOMECS cultivation [3,20], suggesting that VEGF-mediated angiogenesis plays a partial role in the healing of post-ESD esophageal ulcers [20,21]. In addition, human skin fibroblasts exposed to exosomes derived from oral mucosal epithelial cells showed a dose-dependent reduction in proliferation and a considerable increase in the expression of growth factors such as hepatocyte growth factor, VEGF, basic fibroblast growth factor, and connective tissue growth factor [[22], [23], [24]]. However, there have been concerns that the growth factors and angiogenesis caused by AOMECS might play roles not only in wound healing, but also in the growth and metastasis of solid tumors [[23], [25]]. In our study, three cases were diagnosed as SCC after ESD/AOMECS. However, Case 1 showed recurrence after noncurative resection and case 4 was a clearly a metachronous recurrence. Although SCC in case 2 developed at the site of AOMECS, local recurrence was not considered since this case was a curative resection at the first ESD. Whether this case is related to AOMECS requires further investigation.

Because there are no established methods to estimate the long-term safety of AOMECS, including the risk of carcinogenesis, of regenerative medical products in human tissue, this study assessed the status of abnormal DNA damage response in biopsy specimens in situ. The expression pattern of 53BP1 nuclear foci has been reported as a feasible technique to estimate the exaggerated DNA damage response, which can lead to carcinogenesis [6,11]. For example, an increased number and size of 53BP1 foci have been observed in patients with NAFLD and advanced fibrosis, which are risk factors for the development of hepatocellular carcinoma [11]. In addition, colocalization of 53BP1 nuclear focus with the proliferation marker Ki67 is considered a sign of dysregulated DNA damage response, which usually occurs in the resting state in healthy cells. Colocalization of 53BP1 and Ki67 has been reported to increase during esophageal SCC carcinogenesis from non-tumor to intraepithelial neoplasia and then to superficial SCC [6,7]. Our study showed similar trends of increased number and size of 53BP1 foci and colocalization with Ki67 during carcinogenesis. For example, in case 2, a high rate of large foci and colocalization of 53BP1/Ki67 were observed when SCC was confirmed. Accumulation of data is crucial to prove the utility of 53BP1 as a DNA damage marker to track the risk of carcinogenesis after transplantation of AOMECS. It is noteworthy that the expression pattern of 53BP1 was heterogeneous from case to case in our study. Large foci as well as co-localization are signs of dysregulated DNA damage, and both are often present in cancer tissues. However, in AOMECS cases, some non-malignant tissues (second biopsy case 3, first biopsy case 7) presented with a relatively high rate of large foci and co-localization of 53BP1/Ki67. It is unknown if AOMECS contributes to the DNA damage response. However, especially the esophagus undergoes consistent chemical and physical stimulation, often resulting in local inflammation, which may have affected its DNA damage status. We also noted that both specimens were obtained within 3 months after esophageal or pharyngeal ESD. Further comparative studies are required to elucidate the DNA damage status in AOMECS.

In conclusion, our case series suggests that AOMECS yields feasible long-term outcomes after esophageal ESD. The analysis of 53BP1 may reflect internal DNA damage associated with carcinogenesis progression in the esophagus post-ESD/AOMECS as well as DNA damage repair induced by external factors. Continued and meticulous follow-up is crucial to assess the safety of AOMECS transplantation, including potential carcinogenicity.

## Ethics

This study was conducted in compliance with the Declaration of Helsinki and approved by the Institutional Ethical Committees for Medical Research at Nagasaki University (approval no. 18021918). All the human histological samples used in this research had been previously collected and stored after routine examinations during normal medical practice and were not collected specifically and solely for this research.

## Funding statement

This work was supported by a 10.13039/501100001691JSPS Grant-in-Aid for Early-Career Scientists (grant number 20K17024).

## Contribution

Y.M., Y.A., S.K., M.H., and K.H. contributed to the acquisition, analysis, and interpretation of the data and drafting of the manuscript. K.N., Y.S., and N.Z. conducted experiments. N.Y. and M.N. contributed to data acquisition and interpretation. K.N., K.K., and S.E. contributed to the study conception and design; acquisition, analysis, and interpretation of data; and drafted the manuscript.

## Data availability statement

Data are available upon reasonable request.

## Patient consent for publication

Not applicable.

## Declaration of competing interest

The other authors declare no conflicts of interest.
